# MicroRNA‐221 promotes breast cancer resistance to adriamycin via modulation of PTEN/Akt/mTOR signaling

**DOI:** 10.1002/cam4.2817

**Published:** 2020-01-03

**Authors:** Yingchun Yin, Xinmei Wang, Tangyue Li, Qi Ren, Liang Li, Xiaoyu Sun, Baohua Zhang, Xinyun Wang, Hongmei Han, Yangyang He, Zhen Cao, Xiaojie Sun, Ziqiang Zhou

**Affiliations:** ^1^ Department of Pathology The Central Hospital of Zibo Zibo China; ^2^ Shinva Medical Instrument Co, Ltd Zibo China; ^3^ Breast and Thyroid Surgery The Central Hospital of Zibo Zibo China

**Keywords:** adriamycin resistance, Akt/mTOR pathway, breast cancer, microRNA‐221

## Abstract

As a prevalent tumor among women, breast cancer is still an incurable disease due to drug resistance. In this study, we report microRNA‐221 to have a significant effect on breast cancer resistance to adriamycin. The microRNA‐221 is elevated in tumor tissue compared with nearby nontumor samples, as well as in breast cancer cell line with adriamycin resistance (MCF‐7/ADR) compared to its parental line (MCF‐7) and the normal breast epithelial cell line (MCF‐10A). Enforced level of microRNA‐221 promotes cancer resistance to adriamycin, which in turn sustains cell survival and exacerbates malignant formation. Reciprocally, the silence of microRNA‐221 in cancer cells augments the sensitivity to chemotherapy, thereby resulting in enhanced apoptosis of MCF‐7/ADR cells*.* Mechanistically, we identify PTEN as a direct target of microRNA‐221, which was conversely associated with a microRNA‐221 level in breast tumors. The knock‐down of PTEN partially reversed the stimulatory role of microRNA‐221 in the modulation of the Akt/mTOR signaling. Taken together, these findings suggest microRNA‐221 suppresses PTEN transcription and activates Akt/mTOR pathway, which in turn enhances breast cancer resistance to adriamycin and promotes cancer development. Our data thus illuminate the microRNA‐221/PTEN axis may act as a promising strategy for the treatment of chemotherapy‐resistant breast tumors.

## Graphical abstract

The miR‐221 suppresses PTEN transcription and activates Akt/mTOR pathway, which in turn enhances breast cancer resistance to adriamycin and promotes cancer development.

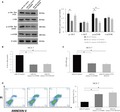

## 1 INTRODUCTION

As a prevalent tumor in women worldwide, the incidence of breast cancer accounts for 29% of all cancers and poses a severe health problem in terms of severe mortality and morbidity.^1^, ^2^ In spite of considerable improvements in therapy, both outcome and prognosis are still barely satisfactory.^3^ Breast cancer cells exhibit elevated proliferative capacity, enhanced invasive and metastatic characteristics, and more seriously, develop the drug resistance during tumor progression. There is a clear consensus that drug resistance of breast cancer is the main obstacle to the improvement of chemotherapy.^4^ However, the process in which cancer cells generate the capacity for drug resistance is still elusive.

It is well documented that microRNAs have a significant effect on the management of various tumor progression.^5^, ^6^ For example, microRNA‐221 is up‐regulated in human oral squamous cell carcinoma, which induces cell proliferation via the modulation of TIMP3 expression.^7^ Moreover, miR‐665 has been reported to promote tumor metastasis by suppression of NR4A3 expression in breast cancer.^8^ However, the function of microRNA‐221 in the modulation of breast carcinoma resistance to chemotherapy is still unknown.

PTEN is recognized as a tumor suppressor gene, which is frequently mutated in multiple human carcinomas and reported to influence cell growth or apoptosis.^9^ Recent studies reveal that miRNA can also regulate PTEN expression. For instance, miR‐130b regulates drug resistance as well as the proliferation of breast cancer cells by targeting PTEN^10^; miR‐21 mediates gemcitabine resistance and epithelial to mesenchymal transition through PTEN/Akt pathway.^11^ The miR‐106b in colorectal cancer plays a role in the regulation of radio‐resistance through the PTEN/PI3K/Akt axis.^12^ However, the effect of microRNA‐221 on adriamycin resistance through the PTEN/Akt/mTOR axis has not been reported.

In this investigation, we found microRNA‐221 was upregulated in breast cancer. Overexpressed microRNA‐221 enhanced cancer resistance to adriamycin, thereby sustaining cell survival and exacerbating malignant formation. Additionally, microRNA‐221 directly inhibits PTEN expression, which in turn activates Akt/mTOR signaling and promotes cell proliferation and invasion. Our study highlighted the role of the microRNA‐221/PTEN axis in drug resistance, which could be exploited for the improvement of chemotherapy against breast cancer.

## 2 MATERIALS AND METHODS

### 2.1 Samples from clinical cases

The examination was performed according to ethical standards. The informed consent as well as the followed investigation has been approved by the Ethics Committee of the Central Hospital of Zibo. After obtaining permission from patients, 25 pairs of breast cancer samples as well as their nearby normal tissues were taken from patients suffering from treatment at the Central Hospital of Zibo from January 2012 to February 2017, and frozen in liquid nitrogen of biobank. All obtained samples were determined to be breast cancer by the pathologist and were immediately frozen for analysis.

### 2.2 Cell preparation and transfection

Breast cancer lines (MCF‐7, MCF‐7/ADR) and human breast epithelial cell line MCF‐10A were gained from the Jiying Biotech. MCF‐10A and MCF‐7 were cultured in DMEM supplemented with 10% fetal bovine serum and 1% penicillin‐streptomycin. To maintain drug resistance, the MCF‐7/ADR cell line was cultured in RPMI 1640 medium (Hyclone) with 1 mg/L adriamycin (Sigma, MO, USA). Cells were plated in a 6‐well plate (Corning, NY, USA) and incubated for 24 hours to reach 80% confluence prior to transfection.

The microRNA‐221 mimic, inhibitor, mimic NC and inhibitor NC were gained from GenePharma (Shanghai, China). For regulation of the miRNA level, the microRNA‐221 in MCF‐7 cells was overexpressed by the transfection of miRNA mimics, and the microRNA‐221 in MCF‐7/ADR cells was knocked down by transfection of microRNA‐221 inhibitor (both performed with Lipofectamine 3000, Invitrogen).

### 2.3 Real‐time quantitative PCR (RT‐qPCR)

Total RNA (including miRNA) was extracted from breast cancer samples or collected cells using the miRNA purification kit (CWBIO). For examination of mRNA expression, the RNA was reversely transcribed into cDNA as instructions of the manufacturer, followed by an examination of RT‐qPCR using SYBR Mix (CWBIO). For the determination of miRNA, the cDNA synthesis reaction was performed with a One‐Step miRNA cDNA Synthesis Kit (including primers for miRNA and U6, HAI‐gene Bio Inc). GAPDH and U6 were indicated as internal controls for mRNA and miRNA, respectively. All samples were examined in triplicate for each specific gene. The primer sequences for PCR are as follows: forward primers 5′‐ TGGATTCGACTTAGACTTGACCT ‐3′ and reverse primer 5′‐ TGCTTTGAATCCAAAAACCTTACT ‐3′ for PTEN, forward primers 5′‐GCCCAATACGACCAAATCC‐3′ and reverse primer 5′‐AGTCCTTCCACGATACCAAAGT‐3′ for GAPDH.

### 2.4 Western blot

Total protein lysates were made in lysis buffer containing 1% PMSF. The concentration of protein in each lysate concentration was quantified by a BCA protein assay kit (CWBIO). Equal proteins from each sample were separated by 10% SDS‐PAGE gels and subsequently transferred to a polyvinylidene difluoride membrane. Membranes were blocked with 5% non‐fat milk and incubated with the primary antibodies against PTEN (Proteintech, 60300‐1‐Ig), p‐Akt (Ser473) (Cell Signaling Technologies, mAb#4060), t‐Akt (Cell Signaling Technologies, mAb#4685), p‐mTOR (Ser2448) (Abcam, ab109268), t‐mTOR (Abcam, ab2732), β‐actin (internal standard, Santa Cruz Biotech, #sc‐47778) overnight, followed by incubation with anti‐rabbit IgG for 2 hours at 37°C. Bands were detected by using the ECL system as the instruction of the manufacturer.

### 2.5 Gene set enrichment analysis (GSEA)

As a specific method to verify if a predefined set of genes showed a statistical difference between two biological states, the GSEA was applied to signal pathway enrichment analysis upon differentially expressed microRNA‐221 in the MCF‐10A transfected with microRNA‐221 mimics and inhibitor. The data of microRNA‐221 were collected and analyzed by a web‐based tool Molecular Signature Database (GDS4797, ://www.broadinstitute.org/gsea/msigdb/annotate.jsp). *P* < .01 was regarded as the symbol to filter the prior defined set of genes.

### 2.6 MTT assays

Cell viability was examined by using the MTT assays according to the manufacturer's instruction. Briefly, after transfection, LY294002 or NVP‐BEZ235 treatment, breast cancer cells were seeded in 96‐well plates at a concentration of 2.5 × 10^4^ cells per well for 24h, followed by incubation with or without adriamycin for 48 hours, respectively. Afterward, we added 20µl MTT (5 mg/mL, 3‐[4,5‐dimethylthiazol‐2‐yl]‐2,5‐diphenyl tetrazolium‐bromide) (Sigma) into each well. All cells were then incubated for 4h at 37°C before the medium was removed. Then, 100 μL DMSO was added to the well and mixed thoroughly to guarantee cytolysis and dissolution of the formazan crystal. The absorbance at 570 nm wavelengths was determined through a microplate reader (Molecular Devices). Three independent assays were performed, and the drug resistance was calculated by comparing the IC50 values (the adriamycin concentration which induced 50% reduction during cell proliferation) from cell growth curves.

### 2.7 Apoptosis assay

Annexin V apoptosis detection Kit (Biotake) was applied to the examination of apoptosis. Briefly, cells from different groups were washed with cold PBS and resuspended in 200 μL of the 1X binding buffer with 10 μL of Annexin V‐FITC. Cells were incubated at 4°C in the dark for 20 minutes and the apoptosis rate was calculated on a Flow Cytometry.

### 2.8 Statistical analysis

Data are represented as mean ± standard deviation (SD) and analyzed by using SPSS V17.0. The Student's t‐test was used for comparing means of two groups and one‐way ANOVA was used for comparing means of multiple groups, followed by Dunnett's *t* test. *P < *.05 was considered significant.

## 3 RESULTS

### 3.1 The microRNA‐221 is increased in breast tumor as well as cell lines

**Figure 1:**
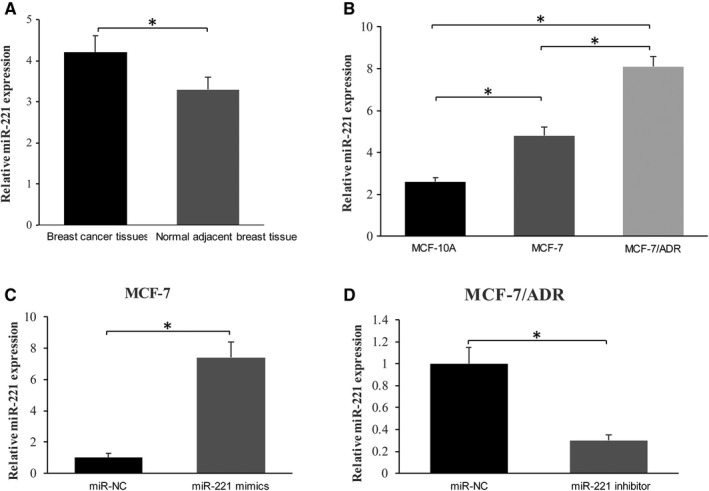
The level of microRNA‐221 in breast cancer and cell lines (A) The level of microRNA‐221 was elevated in breast cancer tissue compared with matched nearby non‐tumor tissue. B, The level of microRNA‐221 in MCF‐7/ADR was higher than any other cell line, and the expression of microRNA‐221 was higher in MCF‐7 cells compared to MCF‐10A cells. C and D, The change of microRNA‐221 expression was listed in MCF‐7, MCF‐7/ADR with transfection of microRNA‐221 mimics/inhibitor/negative control RNA (miRNC). * indicates *P* < .05

To assess the status of microRNA‐221 in breast cancer development, we utilized real‐time ‐PCR to measure the level of microRNA‐221 in samples of patients with breast cancer (Table S1). As shown in Figure 1A, the level of microRNA‐221 was elevated in tumors compared with nearby non‐tumor samples (n = 25/group, *P* < .05). Besides, compared to the MCF‐10A (normal mammary epithelial cell line) and MCF‐7 cells, the MCF‐7/ADR cell line, which is resistant to adriamycin, expresses a higher level of microRNA‐221 (*P* < .05, Figure 1B). To further investigate the effect of microRNA‐221 in the pathogenesis of breast cancer, we used microRNA‐221 mimics or inhibitor to induce overexpression or down‐regulation of microRNA‐221, respectively (Figure 1C,D). Our results thus suggest that increased microRNA‐221 may participate in the physiological activity of breast cancer.

### 3.2 Expression of microRNA‐221 is associated with adriamycin resistance

**Figure 2:**
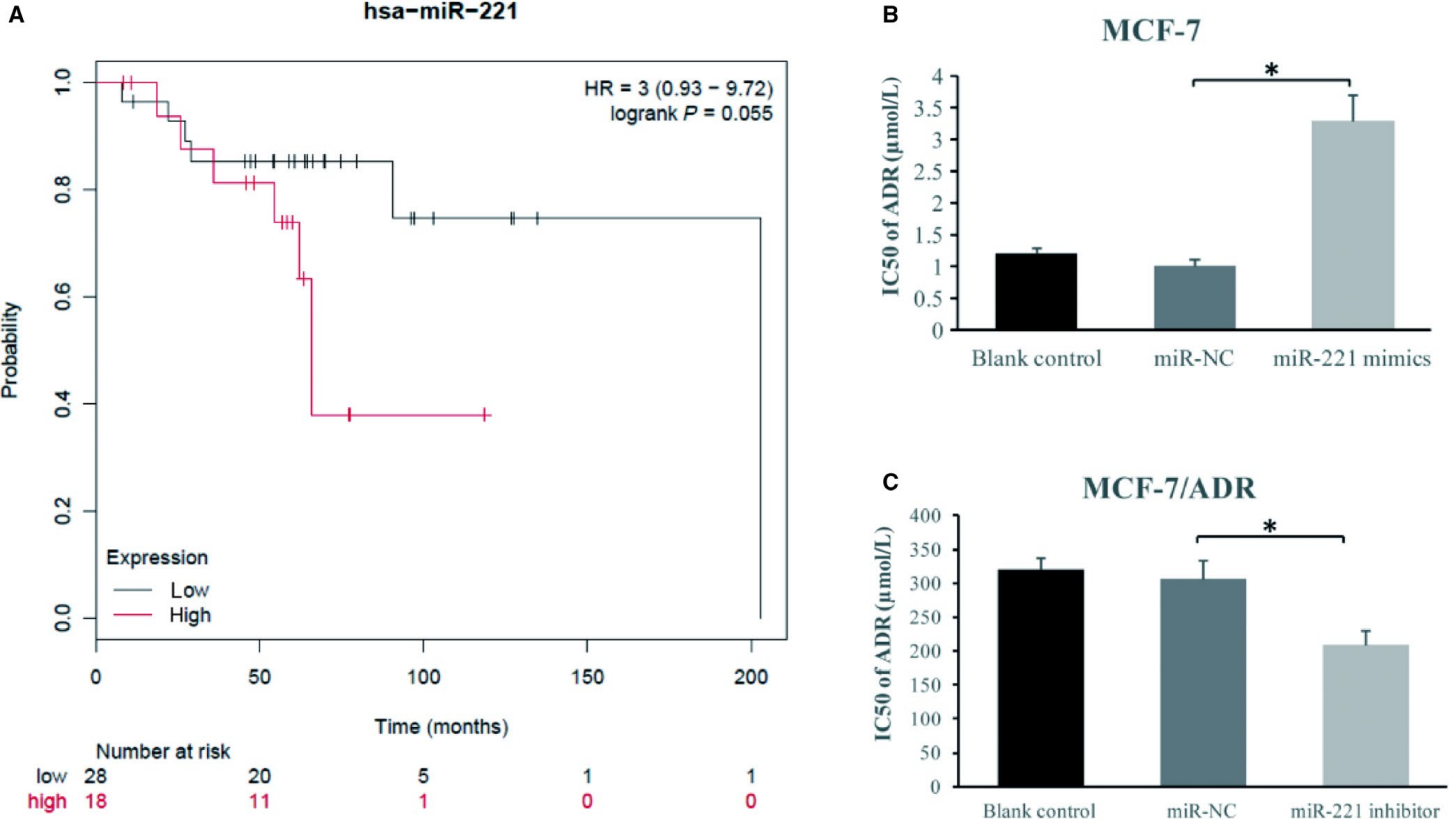
The influence of microRNA‐221 on adriamycin resistance (A) The relationship of overall survival of breast cancer patients in the treatment of chemotherapy and microRNA‐221 expression. Data are from the Kmplot database. B, IC50 of cells transfected with microRNA‐221 mimic highest out of the three groups, and no statistical difference was observed between the blank group and the group transfected with miR‐NC. C, IC50 of adriamycin was lowest in cells transfected with microRNA‐221 inhibitor among the three groups, and no statistical contrast was seen between the blank group and the group transfected with miR‐NC

To investigate the role of microRNA‐221 in cancer progression, we examined the correlation of microRNA‐221 expression with the outcome of patients with breast carcinoma. Unexpectedly, the microRNA‐221 expression is hardly associated with the prognosis of patients with breast cancer. However, patients with a high level of microRNA‐221 exhibited less sensitivity to chemotherapy as relative to those with a low level of microRNA‐221 (Figure 2A, *P* = .055). To further confirm these results, we assessed the sensitivity of MCF‐7 breast cancer cells to adriamycin in the presence or absence of microRNA‐221. As illustrated in Figure 2B, the overexpression of microRNA‐221 obviously enhanced cell survival compared to control groups (1.22 ± 0.09, 1.05 ± 0.12 vs 3.36 ± 0.41, *P* < .05). Furthermore, MCF‐7/ADR cells with transfection of microRNA‐221 inhibitor showed a reduced IC50 of ADR compared with the cells transfected with miR‐NC (320.14 ± 19.03, 307.28 ± 28.42 versus 210.45 ± 20.91 *P* < .05, Figure 2C). Our data thus suggest that upregulated microRNA‐221 in breast cancer is crucial for adriamycin‐resistance.

### 3.3 The microRNA‐221 promotes breast cancer cell survival and proliferation

**Figure 3:**
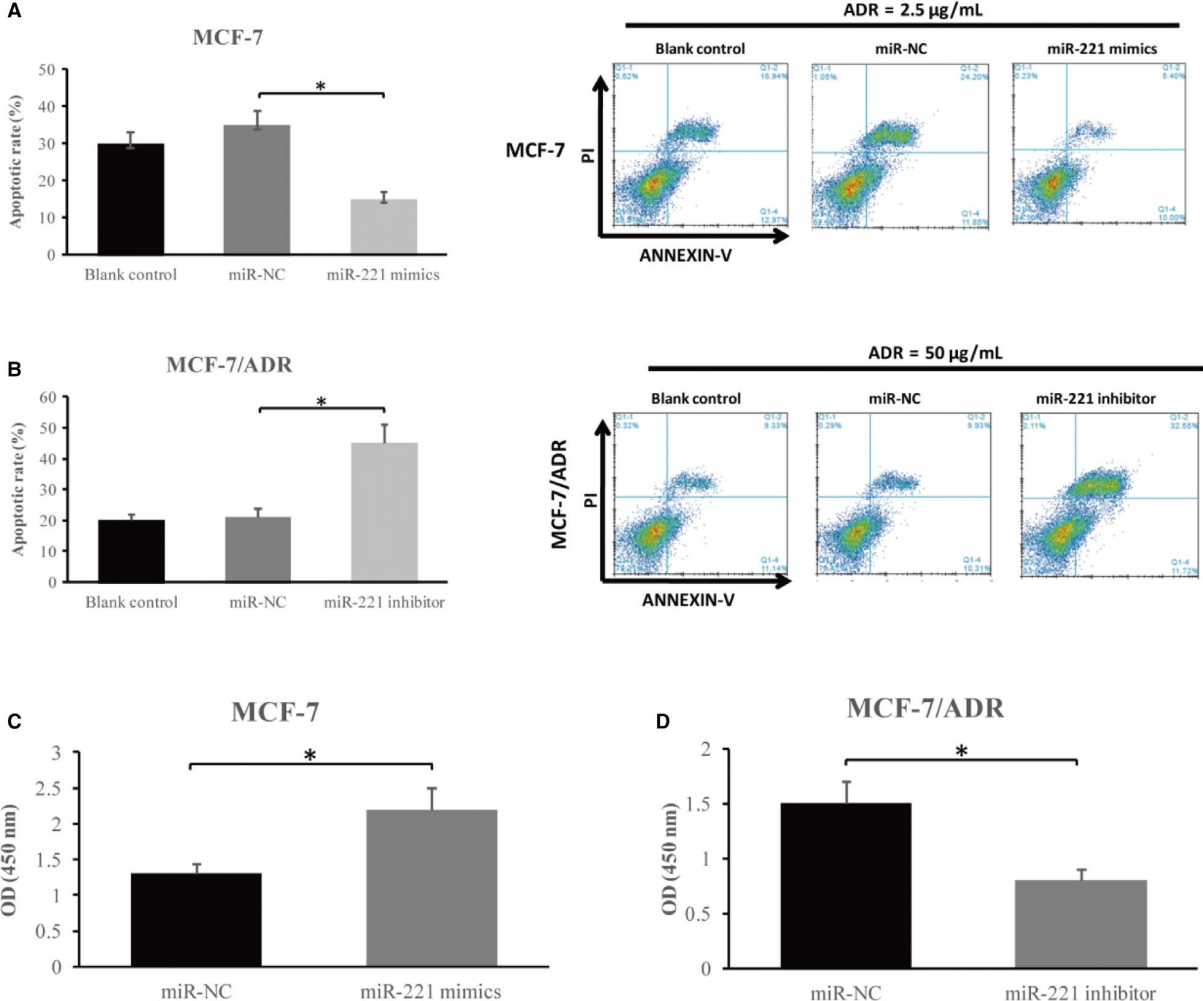
The microRNA‐221 regulates the proliferation and apoptosis of cells (A) MCF‐7 cells were incubated with 2.5 μg/mL of adriamycin for 72 h following transfection of microRNA‐221 mimics. The reduced apoptosis rate was determined by the flow cytometry examination. B, MCF‐7/ADR cells transfected with microRNA‐221 inhibitor led to an up‐regulated apoptosis rate relative to miR‐NC and blank control group. C, The viability of MCF‐7 cells relative to control cells was examined by the MTT method at 72 h. D, The illustration of the proliferation of MCF‐7/ADR cells relative to control cells was determined by the MTT method at 72 h

To verify the biological function of microRNA‐221, we used flow cytometry assay to measure the proliferative or apoptotic capacity of breast cancer cells expressing microRNA‐221. As shown in Figure 3A, the enhanced expression of microRNA‐221 obviously decreased apoptosis in MCF‐7 cells compared to the negative control (*P* < .05). In contrast, down‐regulation of microRNA‐221 led to a significantly elevated apoptotic rate in MCF‐7/ADR cells compared with the control group in the treatment of adriamycin (*P* < .05, Figure 3B).

Cell proliferation was evaluated by the MTT method showed microRNA‐221 increased proliferation in MCF‐7 cells compared with cells transfected with miR‐NC (1.27 ± 0.19 vs 2.21 ± 0.28 *P* < .05, Figure 3C). However, microRNA‐221 silence resulted in a decreased rate of growth compared to the control (1.48 ± 0.23 vs 0.82 ± 0.09 *P* < .05, Figure 3D). Therefore, our results demonstrate that microRNA‐221 is highly associated with adriamycin resistance and augments breast cell proliferation.

### 3.4 The microRNA‐221 regulates the PTEN/Akt/mTOR axis

**Figure 4:**
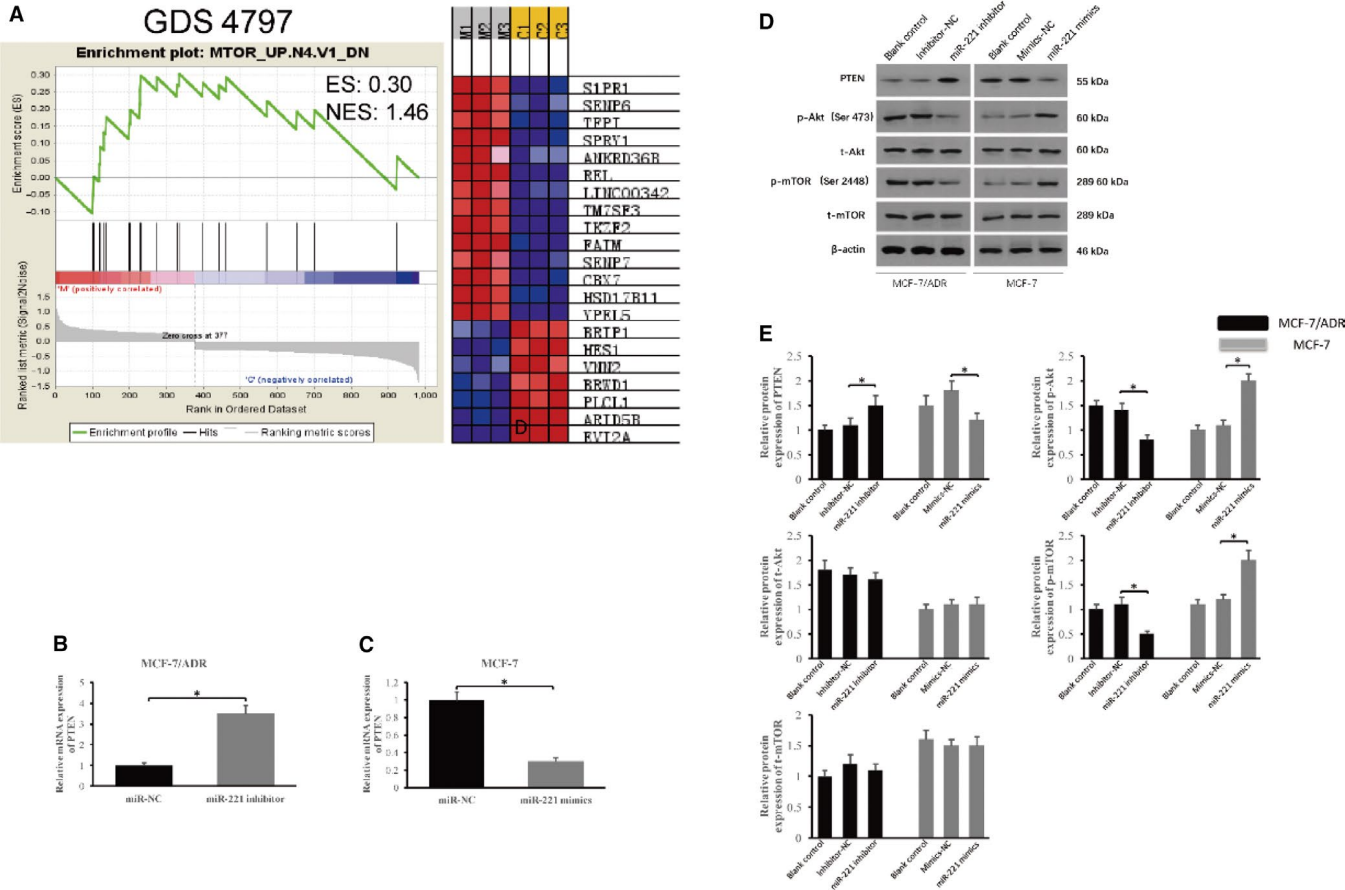
The microRNA‐221 regulates the PTEN/AKT/mTOR axis (A) GSEA of genes differentially expressed in MCF‐10A with or without expression of microRNA‐221 (GDS4797). ES, enrichment score; NES, normalized enrichment score. B, The relative level of PTEN in MCF‐7/ADR cells with the silence of microRNA‐221 and cells transfected with miR‐NC. C, The relative level of PTEN in MCF‐7 cells with over‐expression of microRNA‐221 and cells transfected with miR‐NC. D and E, The influence of microRNA‐221 on the PTEN/AKT/mTOR axis was examined by western blot. Inhibiting microRNA‐221 in MCF‐7/ADR cells promoted expression of PTEN, reduced p‐AKT and p‐mTOR. Overexpressing microRNA‐221 in MCF‐7 cells decreased PTEN, increased p‐AKT and p‐mTOR

To verify the process in which microRNA‐221 modulates cancer resistance to chemotherapy, we interrogated the GSE database and found the GDS4797 containing the differential gene transcription in the presence of microRNA‐221 in the MCF‐10A cell line. Ensued Gene Set Enrichment Analysis (GSEA) showed that microRNA‐221 selectively influenced mTOR signaling activation (Figure 4A). Because PTEN is essential for the regulation of mTOR signaling activation and acts as a potential target of microRNA‐221,^13^ we therefore detected the PTEN expression in presence of microRNA‐221. As illustrated in Figure 4B, the silence of microRNA‐221 led to higher abundance of PTEN transcript in MCF‐7/ADR cells (*P* < .05), while over‐expression of microRNA‐221 suppressed the transcription of PTEN in MCF‐7 cells (*P* < .05, Figure 4C).

To further confirm these results, we performed immunoblots to assess the phosphorylation level of Akt and mTOR in the presence or absence of microRNA‐221. As shown in Figure 4D,E, both p‐Akt and p‐mTOR were elevated in MCF‐7 cells transfected with microRNA‐221 mimics as relative to the miR‐NC, while total Akt and mTOR expression remained identical. Reciprocally, the silence of microRNA‐221 in MCF‐7/ADR led to PTEN upregulation, which in turn suppresses p‐AKT and pm‐TOR, without affecting the expression of total Akt and mTOR (*P* < .05). Our data thus demonstrate that microRNA‐221 can induce Akt/mTOR activation via inhibition of PTEN expression.

### 3.5 Supplementation of Akt/mTOR inhibitor ameliorates adriamycin resistance

**Figure 5:**
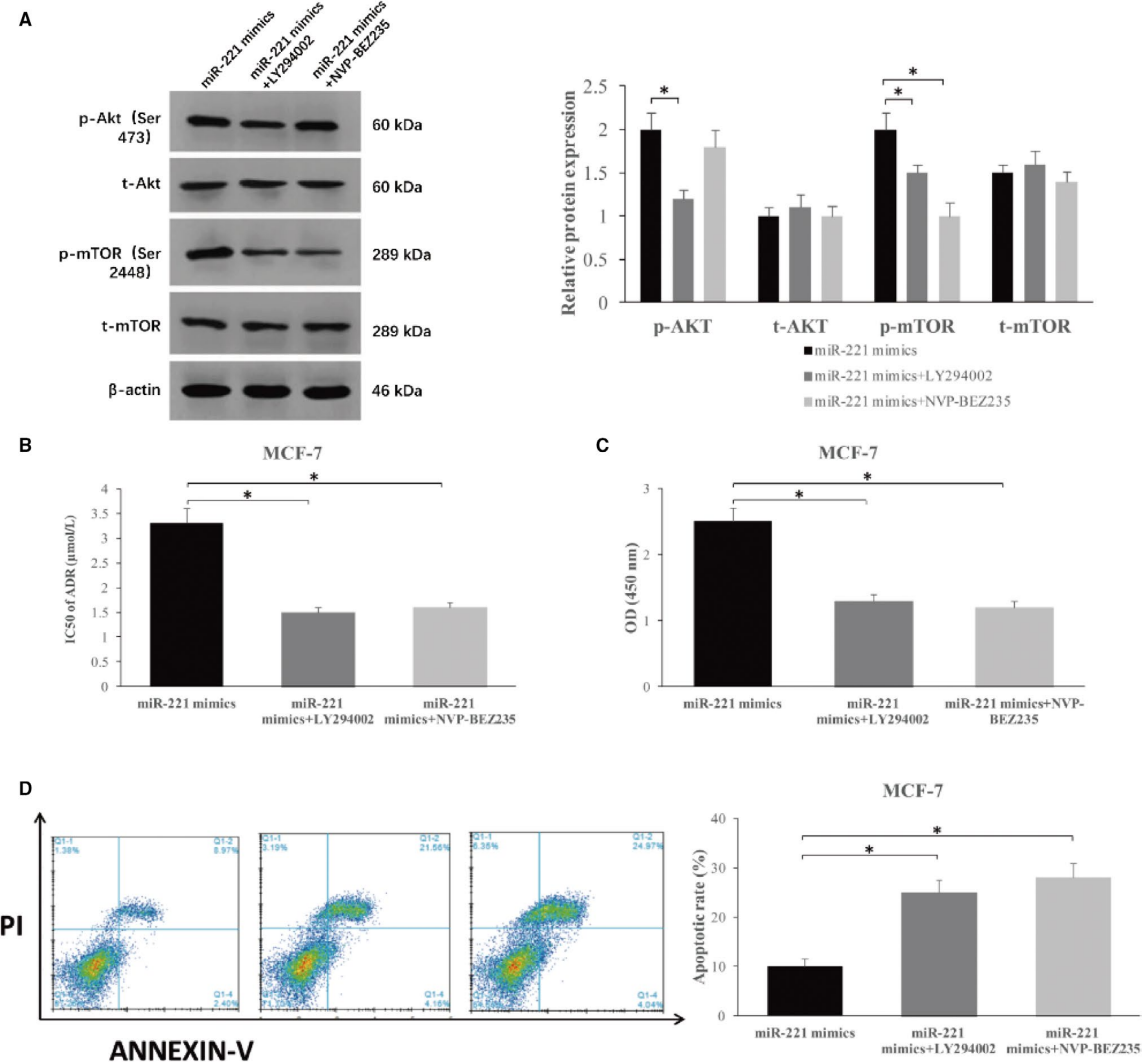
The Akt/mTOR pathway regulates adriamycin resistance in MCF‐7 (A) The effect of LY294002 and NVP‐BEZ235 on PTEN/Akt/mTOR in MCF‐7 overexpressing microRNA‐221 determined by western blot. B, The effect of microRNA‐221 mimics, microRNA‐221 mimics + LY294002 and microRNA‐221 mimics＋NVPBEZ235 on adriamycin resistance of MCF‐7. C, The blockade of the Akt/mTOR axis decreased the proliferation of MCF‐7 cells overexpressing microRNA‐221 detected by MTT assays. D, Inhibitory effect of the Akt/mTOR axis enhanced apoptosis of MCF‐7 cells overexpressing microRNA‐221

To verify whether the stimulatory effects of microRNA‐221 on Akt/mTOR signaling is substantial for chemotherapy resistance, we used LY294002 and NVP‐BEZ235 to block AKT and mTOR signaling transduction. As expected, the enforced level of the phosphorylation of Akt and mTOR induced by microRNA‐221 can be reversed by the treatment of LY294002, while NVP‐BEZ235 treatment selectively inhibited the phosphorylation of mTOR, but not Akt (*P* < .05, Figure 5A). We then treated MCF‐7 cells overexpressing microRNA‐221 with adriamycin with or without Akt/mTOR inhibitors. As shown in Figure 5B, the IC50 of adriamycin was lower in cells with transfection of microRNA‐221 mimics in presence of LY294002 or NVP‐BEZ235 than cells transfected with microRNA‐221 mimics alone, and no difference was seen among cells with miR‐NC, microRNA‐221 mimics plus NVP‐BEZ235, and microRNA‐221 mimics plus LY294002 (3.32 ± 0.31 vs 1.52 ± 0.14, 1.64 ± 0.12 *P* < .05). We thus used MTT and flow cytometry assay to measure the proliferative or apoptotic capacities up exposure to adriamycin. Compared with the control group, both NVP‐BEZ235 and LY294002 treatment exhibited inhibitory effects on the proliferation of MCF‐7 cells overexpressing microRNA‐221 (2.44 ± 0.33 vs 1.21 ± 0.07, 1.14 ± 0.08 *P* < .05, Figure 5C). Moreover, these Akt/mTOR inhibitors enhanced cancer cell sensitivity to adriamycin, thereby leading to a significantly increased ratio of apoptotic cells expressing microRNA‐221 (*P* < .05, Figure 5D). Taken together, our findings demonstrate that microRNA‐221 enhances cancer resistance to adriamycin through suppression of PTEN and activation of Akt/mTOR signaling in breast cancer.

## 4 DISCUSSION

Preventing tumor progression and drug resistance are important goals in the improvement of treatment for breast cancer. Adriamycin is an anthracycline antibiotic commonly used in the therapy of breast cancer, especially in patients with metastatic or recurrent tumors.^14^, ^15^ However, the emergence of resistance to adriamycin poses an enduring obstacle limiting the success of the treatment. Thus, there is an urgent need for strategies to successfully suppress tumor progression, drug resistance, and improve prognosis in breast cancer. Aberrant miRNA expression is ubiquitous in cancer and consequently, can provoke the occurrence and progression of several types of cancer.^5^, ^6^ In our experiments, we investigate the expression and function of microRNA‐221 in breast cancer. Our data suggested that overexpression of microRNA‐221 regulated cell proliferation and conferred resistance to adriamycin via the PTEN/Akt/mTOR axis.

Accumulating evidence demonstrates that miRNAs contribute to the emergence of resistance to chemotherapeutic drugs, such as doxorubicin by regulating various targets in breast cancer.^16^, ^17^ Documents have also shown the role of microRNA‐221 in various types of cancers.^7^, ^18^, ^19^, ^20^, ^21^, ^22^, ^23^ For example, the downregulation of microRNA‐221 in human oral squamous cell carcinoma^7^ increased the sensitivity to adriamycin via regulation of TIMP3 expression. In our experiments, we found that microRNA‐221 was elevated in breast cancer tissue and cell lines, as well as the adriamycin resistance MCF/ADR cells. Moreover, the manipulation of microRNA‐221 expression by transfection affected the tumor cells' adriamycin resistance as well as proliferation in vitro. Meanwhile, knockdown of microRNA‐221 apparently reduced chemoresistance in MCF‐7/ADR cells. These data confirmed that microRNA‐221 takes an oncogenic role in breast cancer cells as the enforcement of microRNA‐221 leads to adriamycin resistance and proliferation while the inhibition of microRNA‐221 via miRNA inhibitor suppresses breast cancer adriamycin resistance and proliferation. Therefore, microRNA‐221 may serve as a promising mediator of resistance to adriamycin and proliferation in breast cancer.

As a widely studied tumor suppressor gene, PTEN has the ability to suppress signal transduction of the Akt/mTOR pathway. Mechanically, at the upstream of Akt, PTEN elicits its phosphatase activity and inhibit the establishment of phosphatidylinositol‐3, 4, 5‐trisphosphate (PIP3) from phosphatidylinositol‐4, 5‐bisphosphate (PIP2), thus antagonizing the activity of PI3 kinase (PI3K) and leading to Akt dephosphorylation.^24^ In recent research, PTEN has been accepted as a target of microRNA‐221, and reported to affect the gefitinib‐resistance of cervical cancer cells.^13^ As for our research, we report that PTEN is crucial for microRNA‐221‐mediated breast cancer resistance to chemotherapy. Our data found that PTEN level was obviously lower in the resistant cells than in the drug‐resistant cell lines. And its expression was inversely regulated by miRNA‐221. Over‐expression of microRNA‐221 decreased PTEN transcript in MCF‐7 cells and silencing microRNA‐221 obviously enhanced PTEN transcript in MCF‐7/ADR cells. These data proved that PTEN participates in chemoresistance, proliferation as well as carcinoma progression.

The Akt/mTOR axis has a significant effect on both physiological and pathological processes, including proliferation, survival, apoptosis, metabolism, which is often constitutively activated in various malignancies.^25^, ^26^ Additionally, unnatural activation of the Akt/mTOR axis is attributed commonly to PTEN disorder, which is one of the frequently mutated tumor suppressors and serves as a crucial negative regulator of this pathway.^27^ Therefore, the PTEN/Akt/mTOR signaling pathway is accepted as a crucial approach in the progression of cancer.^28^ It was reported that overexpression of miR‐222 promoted the sensitivity of bladder cancer cells to drugs and led to cell death by targeting the Akt/mTOR pathway.^29^ Our finding reveals that microRNA‐221 regulated adriamycin resistance in MCF‐7 via the PTEN/Akt/mTOR axis which is in line with previous reports.

In conclusion, our study identifies a newly correlation between the microRNA‐221 level and breast cancer resistance to adriamycin. Through suppression of PTEN expression, microRNA‐221 activates Akt/mTOR signaling and leads to adriamycin insensitivity of breast cancers. These findings warrant further investigation of the underlying mechanisms of adriamycin resistance and may provide an insightful approach for the improvement of treatment for breast cancer.

## CONFLICT OF INTEREST

All authors declare that there are no competing interests associated with the study.
