# Supplementary figures and images for: MicroRNA‐221 promotes breast cancer resistance to adriamycin via modulation of PTEN/Akt/mTOR signaling
Source: Cancer Med. 2020 Jan 3;9(4):1544–52. doi: 10.1002/cam4.2817 (PMC7013069; doi:10.1002/cam4.2817)

**Supplementary Table**

**Table** Clinical information of patients with primary breast tumor


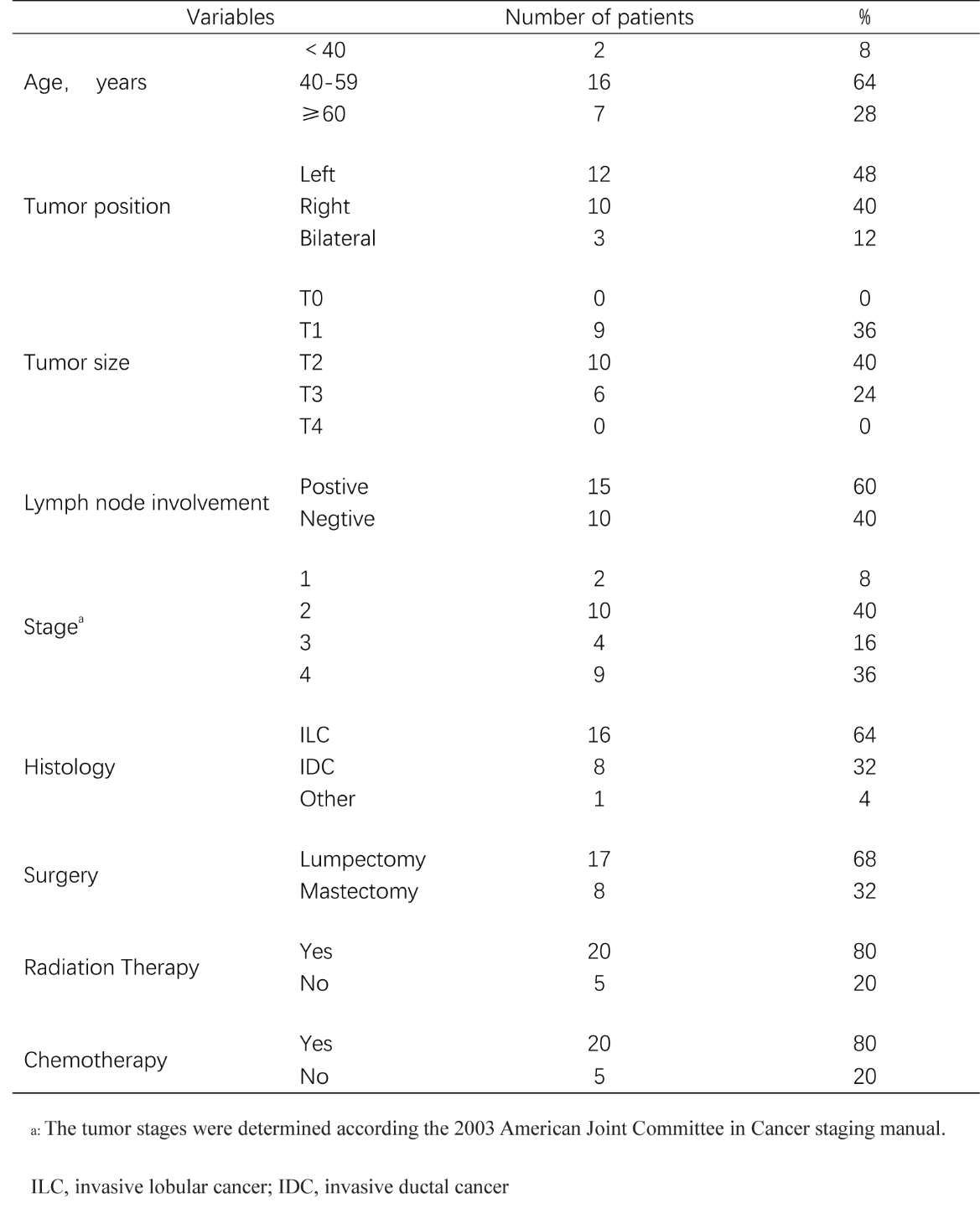

Supplement: Supplementary file 1 [file CAM4-9-1544-s001.docx]
